# Annual Meeting of the Governors and General Council

**Published:** 1918-05-18

**Authors:** 


### KING EDWARD'S HOSPITAL FUND FOR LONDON.

### Annual Meeting of the Governors and General Council.

THE annual meeting of the Governors and General Council of King Edward's Hospital Fund for London, to receive the Accounts and the Report of the General Council for the year 1917, was held at St. James's Palace on Tuesday, May 14, the Speaker of the House of Commons being in the chair.

Those present were: The Viscount Iveagh, the Bishop of London, the Viscount Sandhurst, the Viscount Farquhar, the Lord Revelstoke, the Lord Burnham, the Lord Stamfordham, the Lord Somerleyton (Hon. Secretary), the Lord Stuart of Wortley, the Hon. Sir Arthur Stanley, M.P., the President of the Local Government Board (the Right Hon. W. H. Fisher, M.P.), the President of the Hospital Sunday and Hospital Saturday Funds (the Right Hon. the Lord Mayor), the Chairman of the London County Council (Mr. R. C. Norman), the President of the Royal College of Surgeons (Sir George Makins), the Right Hon. Sir Vezey Strong, Sir William S. Church, Bart., Sir Francis H. Champneys, Bart., Sir Henry Burdett, Sir Horace Marshall, Sir John Craggs, Sir John Tweedy, Mr. Frederick M. Fry (Hon. Secretary), Mr. John G. Griffiths (Hon. Secretary), Mr. Yvon R. Eccles, Dr. Edwin Freshfield, Mr. Geo. Lawson Johnston.

MAINTENANCE OF THE 1917 DISTRIBUTION.

Lord REVELSTOKE (the Hon. Treasurer), in presenting the Account of Receipts and Expenditure and the Balance Sheet for the year ending December 31, 1917, said that he had the honour to submit the Accounts of Receipts and Expenditure and also the Balance Sheet for the year 1917. There was no particular feature to which he thought the attention of the Council should be called. There was certainly no feature of any unsatisfactory nature. On the contrary, it was satisfactory to be able to report that the Fund has been again able to make a substantial increase in the distribution, and also to add to its reserves. If our ordinary income showed no decrease, the Fund should be able to maintain the distribution at or near the total arrived at in 1917.

The SPEAKER OF THE HOUSE OF COMMONS formally moved the adoption of the Accounts.

The resolution, having been seconded by the Viscount IVEAGH, was then put and carried unanimously.

### The Council's Report for 1917.

Mr. FREDERICK M. FRY (Hon. Secretary) presented the draft Report of the Council for the year 1917, as follows:—

1. The total receipts for the year 1917 were £238,572 11s. 9d., of which £1,517 18s. 2d. were contributions to capital, and £237,054 13s. 7d. receipts on general account.

2. General Fund receipts were made up as follows: Annual subscriptions, £24,874 18s. 6d.; donations (including £35,000 from Viscount Astor), £40,206 18s 9d.; League of Mercy, £15,000; estate of Isabella Countess of Wilton, £25,567 10s. 5d.; Lewis estate, £1,000; other legacies, £14,674 3s. 8d.; dividends and interest from investments, £115,531 2s. 3d.; trustees of the Bawden Fund, £200; making a total of £237,054 13s. 7d.

3. The grants made for the year amounted to £190,000, being £20,000 more than in 1916, and £32,500 in excess of the grants made in 1912 and 1913, the years of the highest distribution before the war. The above comparisons do not take into consideration the supplementary distributions made in 1911 and 1915. These were made from special donations by Sir Ernest Cassel, granted after the original distributions were settled.

4. Of the amount distributed, £181,000 was given to London hospitals and £9,000 to consumption sanatoria and convalescent homes taking London patients, as against £162,500 and £7,500 respectively in 1916.

5. The £181,000 granted by the Fund to the London hospitals was applied as to £140,025 in aid of general maintenance, £12,150 to the reduction of debts on maintenance account, and £28,825 towards improvement schemes or in reduction of liabilities on such schemes undertaken before the war. The Fund has continued to encourage the hospitals in the policy of postponing all schemes of capital expenditure not exceptionally urgent or not already in hand at the outbreak of war, and the total grants in aid of new schemes were only £8,675. Grants for maintenance amounted to £8,750 more than in 1916, or £34,650 more than in 1915.

6. This increase in maintenance grants, largely the result of the munificence of Viscount Astor, was fully justified by the evidence as to the needs of the hospitals, which continue to suffer from the rise in prices and other financial difficulties due to the war.

7. Of the £9,000 distributed by the Convalescent Homes Committee, £7,050 was allocated to consumption sanatoria, and £1,950 to convalescent homes. The grants to sanatoria enabled 62 beds to be reserved for the use of patients in London hospitals as compared with 53 last year.

8. The total sum distributed amongst hospitals, convalescent homes and consumption sanatoria during the last ten years (after deducting conditional grants to the extent of £3,500 that have been allowed to lapse) is £1,614,000. Since the foundation of the Fund a total amount of £2,458,416 8s. 5d. has been distributed. The average annual distribution for the whole period of twenty-one years has been £117,067 9s.

9. During the year the amount spent on administration was £3,460 10s., or £1 9s. 0½d. per £100 of the total amount received, as compared with £3,161 2s. 3d., or 19s. 4½d. per £100 in the previous year. The corresponding percentage for the whole period since the inauguration of the Fund has been £1 4s. 6d., or less than 3d. in every £1 received, the average yearly outlay in this respect being £2,865 17s. 3d. The increased expenditure has been occasioned mainly by additions to the remuneration of the staff, and also by the greater cost of stationery, printing, etc., caused by the war.

10. His Majesty the King, Patron of the Fund, has been graciously pleased to continue his annual subscription of £1,000. Her Majesty the Queen, Her Majesty Queen Alexandra, and His Royal Highness the Prince of Wales have also been pleased to subscribe to the Fund, as also have many other members of the Royal Family.

11. The list of contributions again includes annual subscriptions of £5,000 from Mr. Walter Morrison, £4,000 from the Drapers' Company, £1,000 from Lord Astor (in addition to his most generous donation of £35,000), and £1,000 from the Merchant Taylors' Company; besides an anonymous donation of £1,000, and an anonymous gift of War Stock of the same amount. The Fund has also received annual subscriptions of £500 from Lord Iveagh, from Sir John Ellerman, Bt., from Mr. Robert Fleming, from Messrs. Morgan, Grenfell and Company, and from Messrs. N. M. Rothschild and Sons; and donations of the same amount from the Clothworkers' Company, and from "S.D.R.S.D."

**KING EDWARD'S HOSPITAL FUND**—*(continued)*.

12. The League of Mercy this year has contributed £15,000, a sum equal to that which was received from the League last year. The Council is again indebted to the members of the League for the efforts which have led to so successful a result. The total amount contributed to the Fund by the League of Mercy since the foundation in 1899 now amounts to £260,000, apart from the sums awarded by the League to institutions outside the area of the Fund's distributions. The Council again express their appreciation of this very substantial benefit derived from the labours of a numerous band of voluntary workers.

13. Legacies received during the year include £3,712 10s. from the late Field-Marshal Sir Charles Brownlow; £3,893 7s. 1d. on account of the residue of the estate of the late Miss Catherine Hughes; £3,102 11s. 11d., being the balance of the residue of the estate of the late Mr. George Henry Swonnell; £2,000 from the late Mr. Francis Reckitt; a further amount of £1,000 from the executors of the late Mr. Samuel Lewis; and £500 given by the executors of the late Mr. Marcus Van Raalte in the exercise of their discretionary powers.

14. The Fund also received during the year the sum of £25,567 10s. 5d., the balance of the residue of the estate of the late Lady Wilton. This legacy, which has amounted in all to £163,202 9s. 4d., has greatly strengthened the financial position of the Fund at a time when the demands on it are continually increasing.

15. To maintain the annual distribution at £190,000 about £80,000 has to be raised each year from subscriptions, donations, and legacies. Apart from Viscount Astor's donation, the subscriptions and donations received in 1917 were £30,281 17s. 3d., a decrease of £1,158 7s. 8d. as compared with 1916. Legacies decreased from £33,418 7s. 8d. to £14,674 3s. 8d.

16. The Statistical Report on the expenditure of the London hospitals was again issued in its reduced form this year in order to economise the consumption of paper and the employment of labour in printing. From the special table in the prefatory note, it appears that the average number of beds at the London hospitals in daily occupation by naval and military patients in 1916 was 2,855. The total ordinary expenditure, which in 1913 was £1,142,938, had risen in 1916 to £1,513,834, an increase of £370,896. In so far as this increase was due to the treatment of naval or military patients, it was partially covered by contributions from the authorities amounting to £218,087. The balance, amounting to £152,809, represents the additional ordinary expenditure which fell on the funds of the voluntary hospitals of London during 1916; an increase of 13.36 per cent. on the expenditure in 1913. The result has been in several instances a material reduction in the available funds of these most essential institutions.

17. Every eligible hospital which applied for a grant was inspected and reported upon, as hitherto, by two of the Fund's visitors, of whom one was a medical man and the other a layman. Owing to the restrictions on travelling by motor and by rail the inspection of consumption sanatoria had to be suspended. All the sanatoria receiving grants had, however, already been visited for several successive years.

18. The Council regret to record the death during the year of the Duke of Norfolk, one of the original members of the Council and a generous subscriber to its funds. The Fund has also lost through death the services of Sir George Alexander and Mr. Arthur James, who had given valuable help as visitors on behalf of the Fund.

19. Out of the six members of the office staff of the Fund, three have been on active service. The Council have learned with regret that one of these, Mr. Frank G. Simmons, has died of wounds in Palestine, and that another, Mr. Victor H. Rushton, has been wounded and is a prisoner in Germany.

20. The President of the Local Government Board, in pursuance of his powers under the Act, has again appointed Messrs. Deloitte, Plender, Griffiths and Company, chartered accountants, to audit the accounts of the Fund for the year. They have been the Honorary Auditors since January 1898, during which period they have rendered the Fund very valuable service.

21. The Council are under great obligations to the Press for the great assistance they have rendered in the past, and continue to rely upon their kindly co-operation in obtaining for the Fund wider publicity and support.

### Causes for Congratulation.

The Speaker of the House of Commons, in moving the adoption of the Report, said: I need not detain you above a moment in calling attention to one or two matters connected with the Report. The grants amounted to £20,000 more in 1917 than in 1916—a very satisfactory statement. We may also congratulate ourselves on the low cost of management. The amount spent on administration was only £1 9s. 0½d. per £100 of the total amount received, in spite of the increased cost of printing and paper, and the fact that in some cases we are paying the salaries of those of our staff who are on active service. We have again to thank the League of Mercy for a very substantial addition to our funds. I take this opportunity of informing the Council that, for the first time this year, the Governors have secured the services of some ladies, who have been added to the Visitors. This step was not taken without having previously obtained His Majesty's consent, which was readily given. We feel sure that the Fund will be benefited by the assistance of these ladies, who are prepared to undertake work for which they are admirably qualified. I now beg to move the adoption of the Report of the General Council.

The Lord Mayor (Sir Chas. A. Hanson) having seconded the resolution, the adoption of the Report was then put and carried unanimously.

On the motion of the Chairman of the Distribution Committee (Sir William Church), seconded by the President of the Royal College of Surgeons (Sir George Makins), a Report of the Distribution Committee on the grant made in 1917 to the Royal Hospital for Diseases of the Chest was adopted.

Sir Henry Burdett drew attention to the importance of keeping up and increasing the amount received from subscriptions and annual donations; and Lord Somerleyton, on behalf of the Hon. Secretaries, undertook to bring the matter before the Executive Committee.

The President of the Local Government Board (the Right Hon. W. Hayes Fisher, M.P.), in moving a vote of thanks to the Speaker of the House of Commons, said that later in the day the Speaker would be presiding over another Assembly, where a great Budget, dealing with millions of money, would be discussed. The Chancellor of the Exchequer would envy the Council of this Fund its budget dealing with money provided so liberally and expended so well.

The resolution was seconded by the Chairman of the London County Council (Mr. R. C. Norman), and carried unanimously.

The Speaker having briefly acknowledged the vote of thanks, the proceedings terminated.

